# Coping with the toll of child sexual abuse investigations

**DOI:** 10.3389/fpsyg.2025.1584034

**Published:** 2025-08-05

**Authors:** Lauran Easton, John Synnott, Dara Mojtahedi, Maria Ioannou

**Affiliations:** ^1^Merseyside Police, Liverpool, United Kingdom; ^2^Department of Social and Psychological Sciences, University of Huddersfield, Huddersfield, United Kingdom

**Keywords:** policing, child sexual abuse, investigations, coping, wellbeing, ProQOL, depression

## Abstract

**Introduction:**

The policing of child sexual abuse (CSA) can have adverse psychological consequences on police personnel. Though some emerging research has shown a negative impact on quality of life, less is known about the coping strategies used by such professionals when working on CSA cases.

**Methods:**

The present study surveyed 68 police personnel involved in the investigation and/safeguarding of CSA about their occupational wellbeing (Compassion Satisfaction, Burnout, Secondary Traumatic Stress, and Depression) and coping strategies to work-related stress.

**Results:**

Findings demonstrated that the majority of respondents reported low to moderate levels of negative occupational wellbeing, however, a considerable minority, specifically those using venting, self-blame and behavioural disengagement as coping strategies, reported severe negative mental wellbeing.

**Discussion:**

The findings highlight the need for policing organisations to support personnel in identifying and using healthy coping approaches that do not exacerbate the effects workplace stress. The authors identify the need for future research to identify specific problem-focussed approaches that are effective in mitigating the effects of workplace stress on mental wellbeing.

## 1 Introduction

Police officers and staff (herby collectively referred to as *police personnel*) are frequently exposed to operational (e.g., encountering violence and trauma; Abdollahi, 2002, Violanti et al., 2017) and organisational stressors (e.g., poor management, inadequate support and increasing workloads; Jones et al., 2025; Papazoglou et al., 2020; Purba and Demou, 2019). Unsurprisingly, negative mental health is commonplace within policing professions (Burnett et al., 2020; MIND, 2016) with 12% of police officers reporting a lifetime mental health diagnosis and 26% reporting mental illness symptoms (Jetelina et al., 2020). Moreover, police officers are at a greater risk of developing post-traumatic stress disorder (PTSD) (Hartley et al., 2013) and committing suicide compared to the general population (Miller, 2006; Townsend and Savage, 2019). The wider effects of police stress can also impact organisations by impairing productivity (Covey et al., 2013; Wang et al., 2020; Kligyte et al., 2013) and increasing sickness absences (Magnavita and Garabrino, 2013).

In response, police forces have initiated pro-active efforts to foster positive mental health (Phythian et al., 2022; Wild et al., 2020). Within the England and Wales, initiatives such as *Oscar Kilo*, *The Blue Light Wellbeing Framework* and *The National Police Wellbeing Service* have been set up to support the mental health needs of officers. However, as outlined Phythian et al. (2022), there has been limited empirical investigation by external parties into the efficacy of such programmes. Additionally, negative mental health reports have continued to persist since the development of these interventions (Davies et al., 2023) suggesting that greater knowledge on policing mental health is required for interventions to be effective. One domain within policing that has been found to negatively affect the mental health of professionals is the investigation of Child Sexual Abuse (CSA). Though research in this area is limited, emerging studies on police (e.g., Brady, 2017; Tehrani, 2016) and other professionals that engage with CSA cases (e.g., medical professionals, O’Hara et al., 2020; and social workers, Dagan et al., 2016) suggest that the traumatic nature of CSA can be psychologically detrimental for responders. The present study builds on this research by exploring the wellbeing and coping strategies of police personnel who work on CSA cases. The literature review first considers the conceptual frameworks of occupational wellbeing and coping in relation to policing, and then discusses current empirical data around the impact of professional interaction with CSA.

### 1.1 Police personnel’s professional quality of life

The Professional Quality of Life (ProQOL) framework measures the negative and positive effects of helping others who experience suffering and trauma. The framework conceptualises occupational wellbeing as an interplay between Compassion Fatigue and Compassion Satisfaction (Newell and MacNeil, 2011; Stamm, 2005).

#### 1.1.1 Compassion fatigue

Compassion fatigue (CF) reflects the adverse effects that can follow from continuously caring for individuals who are suffering, arising when the demands for compassion outweigh an individual’s capacity (Figley, 1995). The effects of CF include depletion of one’s caring resources, hypervigilance, hopelessness, and concentration deficits, as well as physical and emotional exhaustion (Baverstock and Finlay, 2016; Bride et al., 2007). CF can be broken down further into two adverse occupational reactions: Burnout and Secondary Traumatic Stress (STS; Cocker and Joss, 2016; Stamm, 2005). Burnout represents feelings of emotional exhaustion (i.e., depleted emotional resources), detachment from others (or depersonalisation) and a reduced personal accomplishment that can result from prolonged and untreated occupational stress (Maslach, 1993; World Health Organisation, 2020). STS reflects the negative PTSD-like effects that arise from indirect exposure to trauma (i.e., exposure to the traumatic experience of others; Birch et al., 2017; Bourke and Craun, 2014). While similar in nature, STS and PTSD differ in that PTSD results from direct exposure to trauma and is a formal diagnosis in the DSM-5 (Diagnostic and Statistical Manual of Mental Disorders), whereas STS results from repeated indirect exposure to trauma, usually in the form of traumatic narratives in which symptoms can mimic that of PTSD. Despite this, some evidence suggests that a minority of frontline workers who experience STS also meet diagnostic criteria for PTSD (Bride, 2007).

Since Andersen and Papazoglou (2015) commented on the dearth of literature studying CF within policing, there has been a steady growth in research exploring this phenomenon. Due to the job demands of policing, CF and its components (Burnout and STS) are a recurring occupational hazard for police officers (Battle, 2011; Davies et al., 2023), with approximately one in five police officers exhibiting concerning levels of compassion satisfactions (explained below), burnout and STS (Burnett et al., 2020). This is conceivable given that exposure to critical incidents involving the maltreatment of others can subject police officers to STS (Cross and Ashley, 2004), whilst the immense work demands of police officers also place them at high risk of burnout (Brady, 2017; Kula, 2017; Schaible and Six, 2016; Setti et al., 2016). Unaddressed CF can result in further mental health deterioration, with multiple studies demonstrating that depression among police can be predicted by STS and burnout levels (Davies et al., 2023; Hegney et al., 2014; santa Maria et al., 2018).

Individuals who routinely work on cases involving CSA may be at an even greater risk of developing CF symptoms, due the traumatic nature of such offences. Indirect exposure to CSA has been identified as a cause of CF (Clarke, 2011; Bennett et al., 2005; Conrad and Kellar-Guenther, 2006). Particularly, officers investigating Internet Child Exploitation (ICE) are likely to come across visual evidence of CSA. These investigators play a crucial role in identifying and apprehending abusers who use digital platforms to exploit children (Powell et al., 2014). However, exposure to digital images of CSA places these such individuals at further risk of STS, burnout and CF (Brady, 2017; Krause, 2009; Perez et al., 2010; Tehrani, 2016), with one on four ICE investigators reporting high levels of CF (Brady, 2017). Woodhams and Duran (2024) evidenced the negative impact of exposure to digital child sexual exploitation images (DCSEI) further in a study which found that analytical staff working in media and criminal justice organisations reported significantly higher levels of STS after exposure to visual abuse. Additional effects of DCSEI also include anxiety, depression, intrusive images, and over protection of own children (Burns et al., 2008; Tehrani, 2016).

#### 1.1.2 Compassion satisfaction

In contrast to CF, compassion satisfaction (CS) reflects positive outcomes that arise from helping others (e.g., increased feelings of personal reward, motivation and pleasure; Stamm, 2002). Furthermore, research has demonstrated that CS can foster resilience (Andersen and Papazoglou, 2015) and mitigate the negative effects of CF such as anxiety and depression (Conrad and Kellar-Guenther, 2006; Davies et al., 2023; Hegney et al., 2014; Stamm, 2002).

The consensus around the level of CS within policing is mixed. It is generally accepted that police officers generally display high levels of CS (e.g., Davies et al., 2023). Police personnel investigating CSA may experience compassion satisfaction through protecting victims, securing convictions, or receiving appreciation from families. Such experiences can foster a sense of purpose and resilience, even when faced with repeated exposure to traumatic material. However other research (e.g., Brady, 2017) have observed low levels of CS when specifically focussing on CSA investigators, which could be attributed to the distressing and challenging nature of CSA investigations. Furthermore, CS can coexist with symptoms of burnout or secondary traumatic stress, underscoring the complex nature of occupational wellbeing in CSA work (Brady, 2017; Davies et al., 2023). Acknowledging this duality is essential for developing support frameworks that enhance CS while addressing the risks of compassion fatigue.

### 1.2 Coping responses to occupational stressors

There has been growing interest in how first responders cope with the difficulties of their job (Arble and Arnetz, 2017; Folwell and Kauer, 2018). Individuals will employ various coping methods to minimise aversive reactions associated with negative incidents (Miller and Brown, 2021). Certain coping strategies, such as positive affirmation (Drury et al., 2014), physical and mental self-care efforts (Burnett et al., 2020) and communication with loved ones (Davies et al., 2023) can be effective in protecting individuals from CF. Conversely, other coping efforts that involve avoiding the stressor or negatively appraising the situation can be less effective and lead to the development of significant mental health issues (Hershcovis et al., 2018; Mojtahedi et al., 2024). Problematically, avoidance toward occupational stressors is relatively common amongst police officers who are more likely to use emotional invalidation to subdue work-related stress instead of talking to others due to the stigmatising views toward mental health vulnerability within policing culture (Bell et al., 2022; Davies et al., 2023). This is especially problematic because research indicates that CF is more likely to develop among police officers who ignore occupational stressors and attempt to continue working without seeking support (Papazoglou et al., 2020).

Theoretical understandings of coping have evolved from early dichotomies, such as Lazarus’s (1984) distinction between problem-focussed and emotion-focussed coping, to more nuanced frameworks. In particular, Carver et al. (1989) developed the COPE Inventory to capture a broader range of coping behaviours, which was later refined into the Brief COPE (Carver, 1997) — a more concise tool with 14 theoretically grounded subscales.

The Brief COPE was selected for this study due to its practical brevity, strong psychometric properties, and multidimensional structure. It captures a range of both adaptive and maladaptive coping responses, making it particularly suitable for frontline and trauma-exposed populations such as police personnel. Additionally, it aligns well with the transactional model of stress and coping (Lazarus, 1984), which is relevant for understanding the situational appraisals and regulatory behaviours of individuals working under sustained emotional pressure. Fittingly, previous studies with police and first responders have also used the Brief COPE to measure occupational stress and coping (Acquadro Maran et al., 2015).

Examination of the wider literature suggests that the relationship between certain coping strategies and ProQOL outcomes can differ across different occupations. For instance, among first responders, humour was found to be a problematic coping approach that increased the risk of burnout (Miller and Brown, 2021), whereas among police officers, it has been found to reduce perceived stress of work (Acquadro Maran et al., 2015). Research on police coping strategies indicates that problem-focussed coping strategies (e.g., actively trying to address the situation) are the most used and most effective strategies for protecting an individual from CF whereas the use of avoidant strategies (e.g., emotional disengagement and self-blame) can increase the risk of CF and the development of further mental health issues such as PTSD (Acquadro Maran et al., 2015; Hennig-Fast et al., 2009).

### 1.3 Present study

The aim of the present study was to explore both the psychological impact of professional engagement with CSA and the efficacy of the different coping strategies used by police personnel. This aim was achieved through three interconnected objectives. First, the authors examined the wellbeing of police personnel involved in CSA cases using the ProQOL measures (burnout, STS and CS) and clinical measure of depression (BDI-II) (Objective one). Depression was also included as a measure due to past research identifying it as a comorbid outcome with CF among police officers (Davies et al., 2023; Hegney et al., 2014; Santa Maria et al., 2018; Tehrani, 2016).

Next, the authors examined the prevalence of different coping approaches (outlined by the Brief COPE inventory) among police personnel (Objective two), and finally, the authors examined the relationship between different coping strategies and psychological wellbeing (STS, CS, burnout, and depression) (Objective three).

Based on the previous literature (e.g., Acquadro Maran et al., 2015; Henning-Fast et al., 2009), two directional hypotheses were proposed:

*H1*: Problem-focussed coping strategies (e.g., planning, active coping) will be associated with more positive occupational wellbeing (i.e., lower burnout, STS, and depression; higher CS).

*H2*: Avoidant coping strategies (e.g., behavioural disengagement, denial, substance use) will be associated with poorer occupational wellbeing (i.e., higher burnout, STS, and depression; lower CS).

## 2 Methods

### 2.1 Procedure and design

A cross-sectional design was used to explore the mental wellbeing and coping methods of police personnel involved in CSA cases. An online survey link was emailed to participants and data were collected between September 2020 and February 2021. All participants were first provided with an information sheet that outlined the purpose of the study and the focus of the questions. After providing consent, participants were presented with a battery of questionnaires, these involved demographic and occupational questions, the ProQOL-V questionnaire (Stamm, 2002), the Beck’s Depression Inventory (BDI; Beck et al., 1996), and the BRIEF COPE scale (Carver, 1997).

### 2.2 Respondents

Respondents were police staff and investigators working in an Abusive Images Unit or Sex Offender Unit located in the Northwest of England. The inclusion criteria required participants to have worked on cases involving CSA at these units. An online survey link was initially disseminated by a regional Assistant Chief Constable to local police personnel that had worked on child abuse investigations. A snowball sampling approach was used whereby recipients were asked to forward the survey link to other colleagues who had worked on CSA cases. Ninety-six responses were initially collected; however, 27 cases were removed due to failing to complete any of the psychometric questions^1^. The final sample consisted of 68 participants (*M* age = 43.49, Std. deviation = 6.93), of which 37 were female and 31 were male. Participants had worked in policing for a mean of 17.6 years (Std. deviation = 6.21, range = 3 to 35 years) and 91.2% had over 10 years of experience. Participants worked in roles that either involved the investigation of CSA cases or safeguarding victims from such crimes. Most participants reported working in an investigations department (including evidence collection team) (*n* = 38), other departments identified by the participants were child protection (*n* = 16), child exploitation (*n* = 5), major serious and organised crime (*n* = 5), unity (*n* = 2), modern slavery team (*n* = 1) and a sex offender unit (*n* = 1). Although all participants had engaged with cases of CSA, only 30 (44.1%) had been exposed to CSA imagery. Due to the snowball sampling method and wide distribution chain, it was not possible to determine an exact response rate. However, based on publicly available staffing figures, the final sample of 68 participants may represent approximately one-third to half of the estimated target population, although this figure should be interpreted cautiously.

### 2.3 Materials

Professional Quality of Life Questionnaire (ProQOL5, Stamm, 2002) is a 30-item questionnaire that measures symptoms of CS (*α* = 0.883; e.g., ‘I get satisfaction from being able to help people’.), Burnout (*α* = 0.782; e.g., ‘I am happy’.) and STS (*α* = 0.874; e.g., ‘I think that I might have been affected by the traumatic stress of those I help’.), experienced over the previous 30 days. Participants self-report their experience of each symptom (item) using a five-point scale (1 = ‘never to 5’ = ‘very often’). Some items were slightly reworded to be more applicable to the policing occupations (see Appendix). For example, the item ‘I feel trapped by my job as a helper’ was adapted to ‘I feel trapped by my job working on crimes against children’. Summed scores for each construct can be interpreted as ‘Low’ (<22), ‘Moderate’ (23–41), or ‘High’ (>42) presence of the respective symptom.

The Beck’s Depression Inventory Revised (BDI-II, Beck et al., 1996) is 21-item questionnaire that measures the severity of depression symptoms. The items reflect physical (6 items) or psychological (15 items) symptoms and are scored on four-point scales (e.g., 0 = ‘I do not feel sad’ to 3 = ‘I am so sad and unhappy that I cannot stand it’). Responses are summed to produce a unidimensional score (*α* = 0.928), which are classified as minimal (0–13), mild (14–19), moderate (20–28), or severe (29–63).

The Brief COPE (Carver, 1997) is a shortened version of the COPE inventory (Carver et al., 1989) which measures how frequently individuals use different coping strategies. The 28-item questionnaire measures 14 coping approaches which can be broken down further into problem-focussed coping (active coping, using informational support, positive reframing, and planning), emotion-focussed coping (emotional support, venting, humour, acceptance, religion, self-blame) and avoidant coping (self-distraction, denial, substance abuse, behavioural disengagement). Items are measured on a four-point scale (0 = ‘I have not done this at all’ to 3 = ‘I have done this a lot’) and are averaged with a paired item to calculate a score for each coping style. Participants were asked to respond to each item in relation to dealing with work-related stress. As evidenced in Table 1, most of the coping strategy scales demonstrated strong reliability, with the exception of Self-distraction (*α* = 0.571) and Denial (*α* = 0.491). However, the two low values could be attributed to the scales’ low item count (see Tavakol and Dennick, 2011), thus they were included in the analyses.

**Table 1:** Descriptive statistics, psychometric properties and correlation coefficients for continuous variables.

	1	2	3	4	5	6	7	8	9	10	11	12	13	14	15	16	17	18
1 CS	—																	
2 Burnout	−0.602***	—																
3 STS	−0.276*	0.729***	—															
4 Depres	−0.494***	0.794***	0.768***	—														
5 Active	0.058	0.146	0.162	0.145	—													
6 IS	−0.019	0.156	0.212	0.130	0.674***	—												
7 PR	−0.002	0.011	0.120	0.188	0.561***	0.548***	—											
8 Plan	−0.06	0.318*	0.315*	0.361**	0.735***	0.813***	0.656***	—										
9 ES	0.031	0.091	0.073	0.052	0.591***	0.809***	0.545***	0.638***	—									
10 Venting	−0.266*	0.433***	0.499***	0.473***	0.512***	0.557***	0.390**	0.622***	0.392**	—								
11 Humour	−0.072	0.196	0.087	0.057	0.474***	0.514***	0.459***	0.512***	0.517***	0.280*	—							
12 Accept	0.052	0.272*	0.278*	0.251	0.502***	0.556***	0.478***	0.712***	0.469***	0.520***	0.558***	—						
13 Religion	−0.082	0.208	0.194	0.205	0.545***	0.460***	0.373**	0.550***	0.461***	0.369**	0.415***	0.467***	—					
14 SB	−0.063	0.434***	0.488***	0.531***	0.468***	0.427***	0.485***	0.666***	0.382**	0.451***	0.349**	0.624***	0.419***	—				
15 SD	−0.035	0.245**	0.243	0.247	0.539***	0.375**	0.531***	0.521***	0.416***	0.415***	0.608***	0.545***	0.395**	0.546***	—			
16 Denial	−0.078	0.387**	0.486***	0.454***	0.229	0.219	0.103	0.258	0.182	0.370**	0.080	0.192	0.316*	0.481***	0.155	—		
17 Subs	−0.092	0.312*	0.363***	0.381**	0.369**	0.364**	0.431***	0.431***	0.439***	0.448***	0.314*	0.462***	0.403**	0.313*	0.275*	0.412**	—	
18 BD	0.569***	0.569***	0.540***	0.614***	0.055	0.100	0.086	0.259	0.063	0.315*	0.015	0.286*	0.281*	0.405**	0.212	0.621***	0.437***	—
Mean	34.79	26.43	23.5	13.07	2.57	2.23	2.33	2.57	2.01	1.91	2.36	2.56	1.55	2.42	2.38	1.4	1.6	1.56
Std Dev	6.31	5.84	7.54	10.53	1.03	1.07	1.08	1.08	1.07	1	1.11	1.16	0.94	0.97	1.04	0.66	0.98	0.8
α	0.883	0.782	0.874	0.928	0.756	0.84	0.863	0.828	0.889	0.746	0.862	0.841	0.851	0.609	0.571	0.491	0.958	0.663

*p < 0.05, **p < 0.01, ***p < 0.001.

### 2.4 Analysis

Linear regression analyses were used to see how well coping strategies predicted ProQoL and depression symptoms. Only coping strategy variables that were significantly correlated with the respective outcome variable were included in the models. Whilst this data-driven strategy has been criticised for potentially inflating Type II error and compromising model stability, the approach was deemed suitable for the present study due to reducing the complexity and overfitting of the analysis, especially in small samples (see Babyak, 2004).

Preliminary analyses were conducted to ensure no violation of the assumptions of linearity, and homoscedasticity. The collinearity statistics (VIF & Tolerance) for all models indicated that multicollinearity was unlikely to be a problem (Tolerance >0.1 & VIF < 10 for all predictors; see Tabachnick et al, 2007), although more conservative thresholds (e.g., < 5) are sometimes recommended in small samples.

## 3 Results

### 3.1 Wellbeing of CSA police personnel

Mean and standard deviation scores for the wellbeing outcomes (CS, Burnout, STS and Depression) are presented in Table 1. The nominal scoring guidelines for both inventories were used to contextualise the wellbeing of participants. For Burnout, only one participant (1.5%) reported scores that were considered ‘high’, with the majority of participants reporting ‘average’ (*n* = 52, 76.5%), followed by ‘low’ (*n* = 15, 22.1%) scores. For CS, most participants reported ‘average’ scores of CS (*n* = 57, 83.8%), followed by ‘high’ scores (*n* = 9, 13.2%) and only two participants (2.9%) reported ‘low’ scores. For STS, most participants reported ‘low’ scores (*n* = 38, 55.9%), though a considerable proportion reported ‘average’ scores (*n* = 28, 41.2%) and only two participants (2.9%) reported ‘high’ scores. Together, the findings suggest that while many participants reported moderate levels of burnout or secondary traumatic stress, only a small number exhibited clinically significant symptoms of occupational distress. Of the 51 participants who completed Beck’s Depression Inventory, most reported minimal levels of depression (61.66%, *n* = 37), however 11.6% (*n* = 7) reported mild levels, 16.6% (*n* = 10) reported moderate levels, and 10% (*n* = 6) reported severe levels. The partial completion of the BDI (*n* = 51) may be due to the more sensitive or personal nature of the BDI items, which assess depressive symptoms in greater depth. As such, depression results should be interpreted as representative only of this subsample (*n* = 51), not the entire study population.

### 3.2 Coping strategies of investigators

Next, the authors examined the frequency of different coping strategies used by participants to manage their work-related stress. The mean scores for each coping strategy (see Table 1) suggest that the most frequently used coping strategy by CSA personnel were Active coping (*M* = 2.57, *SD* = 1.03), Planning (*M* = 2.57, *SD* = 1.08), and Acceptance (*M* = 2.56, *SD* = 1.16); the least frequently used coping strategies were Religion (*M* = 1.55, *SD* = 0.94), Denial (*M* = 1.4, *SD* = 0.66), and Behavioural Disengagement (*M* = 1.56, *SD* = 0.80).

Preliminary analyses indicated that the effects of gender, age, policing experience and CSA imagery exposure on the ProQOL and depression measures were non-significant (see Table 2), thus these variables were not included in the regression analyses.

**Table 2:** Effects of gender, image exposure, age and experience on wellbeing outcomes.

	CS	Burnout	STS	Depression
Gender	*t* (66) = 1.25, *p* = 0.214	*t* (66) = −0.34, *p* = 0.735	*t* (66) = −0.76, *p* = 0.452	*t* (58) = −0.84, *p* = 0.404
Indecent image exposure	*t* (66) = 0.66, *p* = 0.255	*t* (66) = 0.68, *p* = 0.25	*t* (66) = 0.09, *p* = 0.464	*t* (58) = 0.23, *p* = 0.41
Age	*r* = 0.044, *p =* 0.725	*r* = −0.081, *p =* 0.521	*r* = −0.031, *p* = 0.806	*r* = −0.035, *p =* 0.791
Experience	*r* = 0.034, *p =* 0.783	*r* = −0.081, *p =* 521	*r* = −0.049, *p* = 0.69	*r* = −0.049 *p* = 0.707

To assess the effectiveness of the coping strategies, four linear regression models were tested to measure the association between coping variables and wellbeing outcomes (CS, Burnout, STS and Depression). The inferential properties for all four models are presented in Table 3. Post-hoc power analyses using G*power 3.1.9.2 (Faul et al., 2009) indicated that the regression models for burnout (*f*^2^ = 0.75, *1*−*β* = 997), STS (*f*^2^ = 0.92, *1*−*β* = 0.999), and Depression (*f*^2^ = 1.09, *1*−*β* = 0.999) were sufficiently powered, however the model for CS (*f*^2^ = 0.11, *1*−*β* = 0.585) was underpowered.

**Table 3:** Inferential properties for regression models (*N* = 56).

	Compassion satisfaction				Burnout				Secondary traumatic stress				Depression			
Variables	*B*	*SE*	β	*t*	*B*	*SE*	β	*t*	*B*	*SE*	β	*t*	*B*	*SE*	β	*t*
Venting	1.26	1.75	0.18	0.72	1.78	0.88	0.31*	2.02	2.91	1.07	0.39**	2.72	2.99	1.41	0.28*	2.13
Behavioural D	−0.99	2.44	−0.12	−0.41	3.6	1.08	0.49**	3.31	3	1.32	0.32*	2.29	5.96	1.74	0.45**	3.44
Planning					−0.31	1.03	−0.06	−0.3	−1.3	1.26	−0.19	−1.03	−1.68	1.57	−0.17	−1.07
Self-blame					1.8	1.12	0.3	1.61	2.92	1.31	0.38*	2.23	3.86	1.62	0.36*	2.39
Denial					−1.19	1.43	−0.14	−0.83	0.1	1.73	0.01	0.06	−1.27	2.2	−0.08	−0.58
Substance abuse					0.13	0.85	0.02	0.15	0.63	1.03	0.08	0.61	0.55	1.31	0.05	0.42
Acceptance					−0.72	0.9	−0.14	−0.79	−1.02	1.08	−0.17	−0.94				
Self-distraction					−0.15	0.8	−0.03	−0.19								

*p < 0.05, **p < 0.01.

The model for CS, composed of two coping strategies that correlated with the outcome variable (Venting and Behavioural disengagement), did not reach statistical significance [*F* (2, 53) = 3; *p* = 0.059, *R^2^* = 0.102], only explaining 10.2% of variance in CS.

The model for Burnout, consisting of eight coping strategies that correlated with the outcome variable (Planning, Venting, Acceptance, Self-blame, Self-distraction, Denial, Substance abuse, Behavioural disengagement), was statistically significant [*F* (8, 55) = 4.44; *p* < 0.001, *R^2^* = 0.430] and explained 43% of variance in the Burnout. The findings suggest that the use of Venting (*β* = 0.31, *p* = 0.049) and Behavioural Disengagement (*β* = 0.49, *p* = 0.002) as coping mechanisms are associated with greater Burnout symptoms.

The model for STS, composed of seven coping strategies correlating with the outcome variable (Planning, Venting, Acceptance, Self-blame, Denial, Substance abuse, Behavioural disengagement), was also statistically significant [*F* (7, 55) = 6.34; *p* < 0.001, *R^2^* = 0.48] and explained 48% of variance in STS. Results suggested that the use of Venting (*β* = 0.39, *p* = 0.009), Self-blame (*β* = 0.38, *p* = 0.03) and Behavioural disengagement (*β* = 0.32, *p* = 0.027) as coping strategies were associated with higher reported levels of STS.

The final regression model for Depression, comprised of six coping strategies that correlated with the outcome variable (Planning, Venting, Self-blame, Denial, Substance abuse, Behavioural disengagement), was also statistically significant [*F* (6, 55) = 8.93; *p* < 0.001, *R^2^* = 0.522] and explained 52.2% of variance in Depression. Results suggest that the use of Venting (*β* = 0.28, *p* = 0.038), Self-blame (*β* = 0.36, *p* = 0.021), and Behavioural disengagement (*β* = 0.45, *p* = 0.001) as coping strategies were associated with higher reported levels of depression. These findings align with theoretical models of maladaptive coping, such as the transactional theory of stress and coping (Lazarus, 1984), which posits that avoidance-based and self-critical coping can exacerbate perceived stress. Behavioural disengagement reflects withdrawal from problem-solving, which has been linked to helplessness and emotional exhaustion (Carver et al., 1989), while self-blame and venting may perpetuate ruminative cycles that intensify negative affect.

## 4 Discussion

Despite some police personnel reporting severe levels of negative mental health, most individuals displayed low levels of STS, burnout, and depression symptoms, and moderate levels of CS. These reports corresponded with ProQOL scores from the wider police populace (e.g., Burnett et al., 2020; Davies et al., 2023), suggesting that police personnel who specifically deal with CSA may not be any more vulnerable to burnout, STS or low CS than their counterparts operating in different divisions. However, due to the relatively small and heterogeneous sample, this conclusion should be interpreted with caution. The variation in roles and exposure levels within the CSA category itself may mask subgroup differences that could emerge in a larger, more stratified sample. Participants displayed lower ProQOL scores in comparison to non-policing child-protection workers from Geoffrion et al. (2019), who scored slightly higher in CS (*M* = 38.06, SD = 5.35), and lower in burnout (*M* = 23.21, SD = 5.04) and STS (*M* = 20.55, SD = 5.35). Taken together, these observations suggest that levels of negative wellbeing among police personnel working on CSA cases are broadly similar to those observed in general policing roles. This may imply that organisational stressors — such as workload pressures, limited managerial support, and resource constraints — play a more pervasive role in shaping occupational wellbeing across divisions than role-specific operational exposures like CSA investigation. However, this interpretation should be treated cautiously and warrants further empirical investigation.

Although the majority of participants from our study reported minimal depressive symptoms, a considerable 38.2% of the sample reported mild to severe symptomology and one in ten participants reported a severe level of symptoms. Average scores on the BDI suggest that depressive symptoms were more prevalent in the current sample (*M* = 13.07) compared to non-policing populations from the same region (Liverpool) which range between 7.9 to 10.7 (see Veerman et al., 2009); the average reported scores from the present study was also higher than reported scores from general police populations (*M* = 9.43, *SD* = 6.32; see Shaffer, 2010), suggesting that police personnel who frequently respond to CSA will present slightly more depressive symptoms.

Average scores on the coping measures did not identify discreet coping preferences by police personnel, however, findings did suggest that certain problem-solving approaches (Active coping and Planning) as well as General Acceptance were more frequent responses to work-related stressors. These findings mirror the reports of police officers from other countries such as Spain (Guerrero-Barona et al., 2021) and Italy (Acquadro Maran et al., 2015), where the same three coping strategies have been identified as the most frequently used. Conversely, both the present study and past research (e.g., Acquadro Maran et al., 2015) suggest that avoidant coping approaches (e.g., denial and behavioural disengagement) are less frequently used by police personnel. Together, these findings suggest that the use of positive coping strategies may be more frequently adopted over maladaptive approaches by police officers, and this appears to hold consistent across different roles and countries – though a more comprehensive cross-national investigation is needed to truly examine how consistent policing coping approaches are across different cultures. Furthermore it is worth noting the self-reported coping approaches may not always be accurate due to the influence of self-serving biases causing some individuals to hold positive illusions about their abilities to cope with stress (see Taylor and Brown, 1988).

Our findings also share some similarity to observations of non-policing professionals who are also exposed to trauma, such as emergency medical service (EMS) workers from Miller and Brown (2021). Both samples identified active coping and acceptance as some of the most frequent approaches used, whilst both reporting denial and behavioural disengagement as some of the least used approaches. There were also some similarities between the current sample and samples of the general public that are not directly exposed to trauma through their occupations such as E-sport gamers from Poulus et al. (2020). That is, both samples reported active coping, planning and acceptance as the most frequently used approaches; both samples also reported religion, denial and behavioural disengagement as the least frequently used coping approaches. However, there were also some key differences, such that policing personnel from the present study appeared to use humour less frequently and substance abuse slightly more often than the general population. These differences could be attributed to the characteristics of stressors each group are exposed to, with humour being an inappropriate response to CSA and alcohol misuse being a more common response to policing stress (Violanti et al., 2011).

The first hypothesis of the study predicted that problem-focussed coping strategies would be associated with positive wellbeing. Although problem-focussed coping approaches were more frequently used, they did not appear to have a noticeable protective effect for individuals against negative wellbeing, thus the first hypothesis was not supported.

The second hypothesis predicted that avoidant-coping strategies would be associated with negative wellbeing. This prediction was partially supported in that behavioural disengagement was significantly associated with burnout, STS and depression. Additionally, emotion-focussed coping strategies of venting and self-blame were also able to predict negative wellbeing outcomes. All three of these approaches reflect a negative reaction to work-related stress that avoid addressing the actual problem. Given the complex nature of CSA, police personnel working on these cases may require support in identifying healthy coping strategies to specific stressors. Whilst it cannot be deduced from the present findings how professional quality of life can be improved for individuals working on CSA cases, it has been evidenced that certain approaches involving avoidance and negative emotional appraisal can have a negative effect. Thus, it is important for policing organisations to engage with personnel and identify strategies for reducing the use of negative coping approaches (e.g., behavioural disengagement) and invest into further research for identifying effective methods for coping with such stressors.

Participants from this study reported some intentions to address the cause of their work-related stress, however the Brief Cope scale did not allow us to determine whether these efforts included external action (i.e., asking for support or discussing the issues with others). Given the longstanding stigmatisation of emotional vulnerability within policing cultures (Bell et al., 2022; Davies et al., 2023), it may be the case that individuals who want to positively address their stressors may attempt to do so alone, which would create further barriers to developing effective coping skills. For this reason, it is important for policing organisations to continue their efforts in creating a culture where emotional help seeking is encouraged, if they want to foster resilience among police personnel.

The interpretation of our findings need to be considered in conjunction with a number of limitations. Firstly, the sample size was comparatively small compared to typical policing survey studies (e.g., Wainwright and Mojtahedi, 2020). Also, whilst all participants had been involved in CSA cases, there were stark differences in the responsibilities of the various departments that participants came from. Due to the specific department groups containing small numbers of respondents, it was not possible to reliably compare the differential impacts of job role on wellbeing or coping approaches. Doing so would have helped identify specific policing tasks that may incur greater aversive reactions and/or maladaptive coping strategies. Future research recruiting a larger sample size should seek to examine differences in wellbeing and coping among these various roles. Further, the methodology and participant recruitment employed in this study may skew the sample toward more connected or engaged officers, potentially under representing the most distressed or isolated officers.

Finally, the study used retrospective survey questions to measure wellbeing and coping strategies. Although adverse emotional experiences can be accurately measured retrospectively (e.g., Conway III et al., 2022), this cross-sectional approach did not allow the authors to establish the causal direction between coping approaches and wellbeing. Future research should employ a longitudinal approach where the coping approaches of police personnel are monitoring in conjunction with changes in wellbeing measures to examine the role of coping methods in mental wellbeing more precisely.

Building on the present findings, the authors propose three directions of academic inquiry. Although, this study did not consider the role of personality in moderating individual’s response to CSA, our findings revealed considerable individual variability in how participants responded to similar operational stressors. Research has demonstrated that dispositional traits can moderate negative affective responses to aversive incidents (e.g., Mental Toughness, see Mojtahedi et al., 2023) and influence individuals’ level of concern and empathy toward victims of sexual exploitation (e.g., right-wing authoritarianism, see Mojtahedi et al., 2024; Stevens et al., 2024). Thus, future research should consider examining the role that personality can play as both a protective factor and risk factor in relation to the police resilience during CSA investigations. The second direction for future research is to examine effective interventions for preventing CF among police personnel working on CSA investigations. Though existing organisation-wide efforts have been implemented in the past, it is important to identify evidence-based strategies that can specifically reduce aversive reactions to encountering CSA. Studies among both police and other populations exposed to traumatic incidents (e.g., prisoners and child social workers) have identified mindfulness interventions as an effective method of reducing negative mental health symptoms (e.g., Brown et al., 2021; Harding-White et al., 2024; Hoeve et al., 2021; Turner et al., 2021); implementation of such strategies within CSA investigation teams could prove to be beneficial. Thirdly, given that the findings suggest that negative wellbeing may be shaped more by organisational than operational stressors, future research should systematically explore the role of organizational dynamics and institutional factors. This includes examining the availability and perceived effectiveness of internal mental health services, organisational culture around help-seeking, peer and supervisory support, and workload management practices in order for successful interventions to be put in place.

## 5 Conclusion

Findings from the present study suggest that most police personnel involved in CSA cases will show little to moderate levels of negative wellbeing which may reflect a degree of resilience among professionals in these roles, although further research is needed to confirm this in larger and more representative samples. These observations should not however be taken lightly, as a notable minority of respondents still reported concerning levels of CS, Burnout, CF and/or Depression, with some of these negative outcomes being exacerbated by maladaptive coping responses. Creating a workplace culture that supports police personnel in adopting effective coping strategies should be an organisational priority, however, to do so, further empirical investment is required to understand the specific sources of stress and the best approaches to mitigating their harm.

## Data Availability

The datasets presented in this article are not readily available because the of the sensitive nature and prearranged confidentiality agreement with participating police organisations. Requests to access the datasets should be directed to the corresponding author who can request permission to share data from the collaborating police organisation.

## Footnotes

^1^Due to the snowball sampling approach, it is not possible to determine the response-rate.

## Appendix

**Table A1:** ProQOL items (* = reworded item).

1 I am happy
2 I am preoccupied with more than one victim.*
3 I get satisfaction from being able to help victims.*
4 I feel connected to others.
5 I jump or am startled by unexpected sounds.
6 I feel invigorated after working with those I help.
7 I find it difficult to separate my personal life from my life at work.*
8 I am not as productive at work because I am losing sleep over the traumatic experiences of a person/case I worked on.*
9 I think that I might have been affected by the traumatic stress of victims I helped in the past.*
10 I feel trapped by my job working with crimes against children/indecent images of children*
11 Because of my job, I have felt “on edge” about various things.
12 I like my work.
13 I feel depressed because of the traumatic experiences of the people I’ve helped.
14 I feel as though I am experiencing the trauma of someone I have helped.
15 I have beliefs that sustain me.
16 I am pleased with how I am able to keep up with working techniques and protocols.
17 I am the person I always wanted to be.
18 My work makes me feel satisfied.
19 I feel worn out because of my work.
20 I have happy thoughts and feelings about victims I have helped.*
21 I feel overwhelmed because my cases and my workload seems endless.*
22 I believe I can make a difference through my work.
23 I avoid certain activities or situations because they remind me of frightening experiences of the people I have helped
24 I am proud of what I can do to help
25 As a result of my work, I have intrusive, frightening thoughts.
26 I feel “bogged down” by the system.
27 I have thoughts that I am a “success” at my job.*
28 I cannot recall important parts of my work with trauma victims.
29 I am a very caring person.
30 I am happy that I chose to do this work.

## References

[ref1] Abdollahi M. K. (2002). Understanding police stress research. J. Forensic Psychol. Pract. 2, 1–24.

[ref2] Acquadro Maran D. Varetto A. Zedda M. Ieraci V. (2015). Occupational stress, anxiety and coping strategies in police officers. Occup. Med. 65, 466–473. doi: 10.1093/occmed/kqv060, PMID: 26048331

[ref3] Andersen J. P. Papazoglou K. (2015). Compassion fatigue and compassion satisfaction among police officers: an understudied topic. Int. J. Emerg. Mental Health Human Resilience 17, 661–663.

[ref4] Arble E. Arnetz B. B. (2017). A model of first-responder coping: an approach/avoidance bifurcation. Stress. Health 33, 223–232. doi: 10.1002/smi.2692, PMID: 27500991 PMC6525630

[ref5] Babyak M. A. (2004). What you see may not be what you get: a brief, nontechnical introduction to overfitting in regression-type models. Psychosom. Med. 66, 411–421. doi: 10.1097/01.psy.0000127692.23278.a9, PMID: 15184705

[ref6] Battle L. (2011). Compassion fatigue, compassion satisfaction, and burnout among police officers who have experienced previous perceived traumas. Univ Memphis 17, 661–663.

[ref7] Baverstock A. C. Finlay F. O. (2016). Maintaining compassion and preventing compassion fatigue: a practical guide. Arch. Dis. Child. Educ. Pract. Ed. 101, 170–174. doi: 10.1136/archdischild-2015-308582, PMID: 26510446

[ref9002] Beck A. T. Steer R. A. Brown G. K. (1996) BDI-II: Beck Depression Inventory Manual. 2nd ed. San Antonio: Psychological Corporation

[ref9011] Bell S. Palmer-Conn S., and Kealey N. (2022). ‘Swinging the lead and working the head’–An explanation as to why mental illness stigma is prevalent in policing. The Police Journal, 95, 4–23. doi: 10.1177/0032258X211049009

[ref8] Bennett S. Plint A. Clifford T. J. (2005). Burnout, psychological morbidity, job satisfaction, and stress: a survey of Canadian hospital based child protection professionals. Arch. Dis. Child. 90, 1112–1116. doi: 10.1136/adc.2003.048462, PMID: 16243862 PMC1720159

[ref10] Birch P. Vickers M. H. Kennedy M.. (2017). Wellbeing, occupational justice and police practice: an ‘affirming environment. Police Pract. Res. 18, 26–36. doi: 10.1080/15614263.2016.1205985

[ref11] Bourke M. L. Craun S. W. (2014). Secondary traumatic stress among internet crimes against children task force personnel: impact, risk factors and coping strategies. Sex. Abuse 26, 586–609. doi: 10.1177/1079063213509411, PMID: 24259539

[ref12] Brady P. Q. (2017). Crimes against caring: exploring the risk of secondary traumatic stress, burnout, and compassion satisfaction among child exploitation investigators. J. Police Crim. Psychol. 32, 305–318. doi: 10.1007/s11896-016-9223-8

[ref14] Bride B. E. (2007). Prevalence of secondary traumatic stress among social workers. Soc. Work 52, 63–70. doi: 10.1093/sw/52.1.63, PMID: 17388084

[ref15] Bride B. E. Radey M. Figley C. R. (2007). Measuring compassion fatigue. Clin. Soc. Work. J. 35, 155–163. doi: 10.1007/s10615-007-0091-7

[ref16] Brown S. M. Bender K. A. Bellamy J. L. Garland E. L. Dmitrieva J. Jenson J. M. (2021). A pilot randomized trial of a mindfulness-informed intervention for child welfare-involved families. Mindfulness 12, 420–435. doi: 10.1007/s12671-018-1001-5

[ref17] Burnett M. E. Sheard I. St Clair-Thompson H. (2020). The prevalence of compassion fatigue, compassion satisfaction and perceived stress, and their relationships with mental toughness, individual differences and number of self-care actions in a UK police force. Police Pract. Res. 21, 383–400. doi: 10.1080/15614263.2019.1617144

[ref18] Burns C. M. Morley J. Bradshaw R. Domene J. (2008). The emotional impact on and coping strategies employed by police teams investigating internet child exploitation. Traumatology 14, 20–31. doi: 10.1177/1534765608319082

[ref19] Carver C. S. (1997). You want to measure coping but your protocol’s too long: consider the brief COPE. Int. J. Behav. Med. 4, 92–100. doi: 10.1207/s15327558ijbm0401_6, PMID: 16250744

[ref22] Carver C. S. Scheier M. F. Weintraub J. K. (1989). Assessing coping strategies: a theoretically based approach. J. Pers. Soc. Psychol. 56, 267–283. doi: 10.1037/0022-3514.56.2.267, PMID: 2926629

[ref23] Clarke J. (2011). Working with sex offenders: best practice in enhancing practitioner resilience. J. Sex. Aggress. 17, 335–355. doi: 10.1080/13552600.2011.583781

[ref24] Cocker F. Joss N. (2016). Compassion fatigue among healthcare, emergency and community service workers: a systematic review. Int. J. Environ. Res. Public Health 13:618. doi: 10.3390/ijerph13060618, PMID: 27338436 PMC4924075

[ref25] Conrad D. Kellar-Guenther Y. (2006). Compassion fatigue, burnout, and compassion satisfaction among Colorado child protection workers. Child Abuse Negl. 30, 1071–1080. doi: 10.1016/j.chiabu.2006.03.009, PMID: 17014908

[ref26] Conway L. G. III Woodard S. R. Zubrod A. Tiburcio M. Martínez-Vélez N. A. Sorgente A.. (2022). How culturally unique are pandemic effects? Evaluating cultural similarities and differences in effects of age, biological sex, and political beliefs on COVID impacts. Front. Psychol. 13:937211. doi: 10.3389/fpsyg.2022.937211, PMID: 36600725 PMC9807227

[ref28] Covey T. J. Shucard J. L. Violanti J. M. Lee J. Shucard D. W. (2013). The effects of exposure to traumatic stressors on inhibitory control in police officers: a dense electrode array study using a go/no-go continuous performance task. Int. J. Psychophysiol. 87, 363–375. doi: 10.1016/j.ijpsycho.2013.03.009, PMID: 23528305

[ref29] Cross C. L. Ashley L. (2004). Police trauma and addiction: coping with the dangers of the job. FBI L. Enforcement Bull 73:24.

[ref30] Dagan S. W. Ben-Porat A. Itzhaky H. (2016). Child protection workers dealing with child abuse: the contribution of personal, social and organizational resources to secondary traumatization. Child Abuse Negl. 51, 203–211. doi: 10.1016/j.chiabu.2015.10.008, PMID: 26549769

[ref31] Davies L. E. Brooks M. Braithwaite E. C. (2023). Compassion fatigue, compassion satisfaction, and burnout, and their associations with anxiety and depression in UK police officers: a mixed method analysis. Police J 96, 509–529. doi: 10.1177/0032258X221106107

[ref34] Drury V. Craigie M. Francis K.. (2014). Compassion satisfaction, compassion fatigue, anxiety, depression and stress in registered nurses in Australia: phase 2 results. J. Nurs. Manag. 22, 519–531. doi: 10.1111/jonm.12168, PMID: 24926496

[ref35] Faul F. Erdfelder E. Buchner A. Lang A.-G. (2009). Statistical power analyses using G*power 3.1: tests for correlation and regression analyses. Behav. Res. Methods 41, 1149–1160. doi: 10.3758/BRM.41.4.1149, PMID: 19897823

[ref36] Figley C. R. (1995). Compassion fatigue: Coping with secondary traumatic stress disorder in those who treat the traumatised. New York, NY: CRC Press.

[ref37] Folwell A. Kauer T. (2018). ‘You see a baby die and you’re not fine:‘a case study of stress and coping strategies in volunteer emergency medical technicians. J. Appl. Commun. Res. 46, 723–743. doi: 10.1080/00909882.2018.1549745

[ref9004] Geoffrion S. Lamothe J. Morizot J. Giguère C. É. (2019). Construct validity of the professional quality of life (ProQoL) scale in a sample of child protection workers. Journal of traumatic stress, 32, 566–576. doi: 10.1002/jts.22410 31265178 PMC6771892

[ref39] Guerrero-Barona E. Guerrero-Molina M. Chambel M. J. Moreno-Manso J. M. Bueso-Izquierdo N. Barbosa-Torres C. (2021). Suicidal ideation and mental health: the moderating effect of coping strategies in the police force. Int. J. Environ. Res. Public Health 18:8149. doi: 10.3390/ijerph18158149, PMID: 34360441 PMC8345933

[ref42] Harding-White M. Mojtahedi D. Carson J. (2024). Group-based mindfulness interventions in prisons: a selective critical review. J. Forensic Prac. 26, 1–17. doi: 10.1108/JFP-10-2022-0054

[ref43] Hartley T. A. Violanti J. M. Sarkisian K. Andrew M. E. Burchfiel C. M. (2013). PTSD symptoms among police officers: associations with frequency, recency, and types of traumatic events. Int. J. Emerg. Ment. Health 15, 241–253, PMID: 24707587 PMC4734407

[ref44] Hegney D. G. Craigie M. Hemsworth D.. (2014). Compassion satisfaction, compassion fatigue, anxiety, depression and stress in registered nurses in Australia: study 1 results. J. Nurs. Manag. 22, 506–518. doi: 10.1111/jonm.12160, PMID: 24175955

[ref45] Hennig-Fast K. Werner N. S. Lermer R. Latscha K. Meister F. Reiser M.. (2009). After facing traumatic stress: brain activation, cognition and stress coping in policemen. J. Psychiatr. Res. 43, 1146–1155. doi: 10.1016/j.jpsychires.2009.03.001, PMID: 19358996

[ref46] Hershcovis M. S. Cameron A. F. Gervais L. Bozeman J. (2018). The effects of confrontation and avoidance coping in response to workplace incivility. J. Occup. Health Psychol. 23:163. doi: 10.1037/ocp0000078, PMID: 28191998

[ref47] Hoeve M. de Bruin E. I. van Rooij F. Bögels S. M. (2021). Effects of a mindfulness-based intervention for police officers. Mindfulness 12, 1672–1684. doi: 10.1007/s12671-021-01631-7

[ref49] Jetelina K. K. Molsberry R. J. Gonzalez J. R.. (2020). Prevalence of mental illness and mental health care use among police officers. JAMA Netw. Open 3, –e2019658. doi: 10.1001/jamanetworkopen.2020.19658, PMID: 33026452 PMC7542299

[ref50] Jones M. Mojtahedi D. West A. (2025). Child abduction murder investigations: an exploration of investigative barriers from the perspective of the senior investigating officer. Policing: an. Int. J. 48, 630–647. doi: 10.1108/PIJPSM-07-2024-0117

[ref51] Kligyte V. Connelly S. Thiel C. Davenport L. (2013). The influence of anger, fear, and emotional regulation on ethical decision making. Hum. Perform. 26, 297–326. doi: 10.1080/08959285.2013.814655

[ref52] Krause M. (2009). Identifying and managing stress in child pornography and child exploitation investigators. J. Police Crim. Psychol. 24, 22–29. doi: 10.1007/s11896-008-9033-8

[ref53] Kula S. (2017). Occupational stress, supervisor support, job satisfaction, and work-related burnout: perceptions of Turkish National Police (TNP) members. Police Pract. Res. 18, 146–159. doi: 10.1080/15614263.2016.1250630

[ref56] Lazarus R. S. (1984). Stress, appraisal, and coping, vol. 464. New York: Springer.

[ref57] Magnavita N. Garabrino S. (2013). Is absence related to work stress? A repeated cross-sectional study on a special police force. Am. J. Ind. Med. 56, 765–775. doi: 10.1002/ajim.22155, PMID: 23334868

[ref58] Maslach C. (1993). “Burnout: a multidimensional perspective” in Series in applied psychology: Social issues and questions. Professional burnout: Recent developments in theory and research. eds. Schaufeli W. B. Maslach C. Marek T. (Philadelphia, PA, US: Taylor & Francis), 19–32.

[ref59] Miller L. (2006). Practical police psychology: Stress management and crisis intervention for law enforcement. Springfield, IL: Charles C Thomas.

[ref60] Miller A. Brown L. (2021). Coping mechanism and professional quality of life in Northeast Texas EMS personnel during the COVID-19 pandemic: an exploratory study. Aust. J. Paramed. 18, 1–8. doi: 10.33151/ajp.18.925

[ref61] MIND (2016) Wellbeing and mental health support in the emergency services. Available online at: https://www.mind.org.uk/media-a/4524/20046_mind-blue-light-programme-legacy-report-v12_online.pdf (accessed 17 September 2024)

[ref62] Mojtahedi D. Dagnall N. Denovan A. Clough P. Dewhurst S. Hillier M.. (2023). Competition anxiety in combat sports and the importance of mental toughness. Behav. Sci. 13:713. doi: 10.3390/bs13090713, PMID: 37753991 PMC10525228

[ref63] Mojtahedi D. Stevens K. L. Austin A. (2024). Right-wing ideology fuels bias against sex trafficking victims: the mediating role of sexism. Psychol. Crime Law. 1–25. doi: 10.1080/1068316X.2024.2386557

[ref65] Newell J. M. MacNeil G. A. (2011). A comparative analysis of burnout and professional quality of life in clinical mental health providers and health care administrators. J. Work. Behav. Health 26, 25–43. doi: 10.1080/15555240.2011.540978

[ref9001] O’Hara M. A. McCann T. A. Fan W. Lane M. M. Kernie S. G. Rosenthal S. L. (2020). Child abuse taking its toll on the emotional well-being of pediatricians. Clinical pediatrics, 59, 450–457. doi: 10.1177/0009922820905865 32070135

[ref67] Papazoglou K. Marans S. Keesee T.. (2020). Police compassion fatigue. FBI Law Enforce. Bull., 1–9.

[ref68] Perez L. M. Jones J. Englert D. R. Sachau D. (2010). Secondary traumatic stress and burnout among law enforcement investigators exposed to disturbing media images. J. Police Crim. Psychol. 25, 113–124. doi: 10.1007/s11896-010-9066-7

[ref69] Phythian R. Birdsall N. Kirby S.. (2022). Developments in UK police wellbeing: a review of blue light wellbeing frameworks. Police J. 95, 24–49. doi: 10.1177/0032258X211073003

[ref70] Poulus D. Coulter T. J. Trotter M. G. Polman R. (2020). Stress and coping in esports and the influence of mental toughness. Front. Psychol. 11:628. doi: 10.3389/fpsyg.2020.00628 32390900 PMC7191198

[ref71] Powell M. B. Cassematis P. Benson M. S. Smallbone S. Wortley R. (2014). Police officers' perceptions of the challenges involved in internet child exploitation investigation. Policing 37, 543–557. doi: 10.1108/PIJPSM-08-2013-0080

[ref72] Purba A. Demou E. (2019). The relationship between organisational stressors and mental wellbeing within police officers: a systematic review. BMC Public Health 19, 1–21. doi: 10.1186/s12889-019-7609-0 31615479 PMC6792329

[ref74] Santa Maria A. Wörfel F. Wolter C. Gusy B. Rotter M. Stark S.. (2018). The role of job demands and job resources in the development of emotional exhaustion, depression, and anxiety among police officers. Police Q. 21, 109–134. doi: 10.1177/1098611117743957

[ref75] Schaible L. M. Six M. (2016). Emotional strategies of police and their varying consequences for burnout. Police Q. 19, 3–31. doi: 10.1177/1098611115604448

[ref76] Setti I. Lourel M. Argentero P. (2016). The role of affective commitment and perceived social support in protecting emergency workers against burnout and vicarious traumatization. Traumatology 22:261. doi: 10.1037/trm0000072

[ref9005] Shaffer T. J. (2010). A comparison of firefighters and police officers: the influence of gender and relationship status. Adultspan Journal, 9, 36–49. doi: 10.1002/j.2161-0029.2010.tb00070.x

[ref79] Stamm B. H. (2002). “Measuring compassion satisfaction as well as fatigue: developmental history of the compassion satisfaction and fatigue test” in Treating compassion fatigue. ed. Figley C. R. (New York: Brunner-Routledge), 107–119.

[ref80] Stamm B. H. (2005). The ProQOL manual: The professional quality of life scale: Compassion satisfaction, burnout and compassion fatigue/secondary trauma scales. Baltimore, MD: Sidran Press.

[ref81] Stevens K. Mojtahedi D. Austin A. (2024). Juror decision-making within domestic sex trafficking cases: do pre-trial attitudes, gender, culture and right-wing authoritarianism predict believability assessments? J. Crim. Psychol. 14, 240–258. doi: 10.1108/JCP-09-2023-0059

[ref9003] Tabachnick B. G. Fidell L. S. Ullman J. B. (2007). Using multivariate statistics (Vol. 5, No. 7). Boston, MA: pearson.

[ref83] Tavakol M. Dennick R. (2011). Making sense of Cronbach's alpha. Int. J. Med. Educ. 2:53. doi: 10.5116/ijme.4dfb.8dfd, PMID: 28029643 PMC4205511

[ref84] Taylor S. E. Brown J. D. (1988). Illusion and well-being: a social psychological perspective on mental health. Psychol. Bull. 103, 193–210. doi: 10.1037/0033-2909.103.2.193 3283814

[ref85] Tehrani N. (2016). Extraversion, neuroticism and secondary trauma in internet child abuse investigators. Occup. Med. 66, 403–407. doi: 10.1093/occmed/kqw004, PMID: 26928859 PMC4913363

[ref86] Townsend M. Savage M. (2019). Labour calls for review into police welfare as suicide figures revealed. London, UK: The Guardian, 22.

[ref88] Turner M. King N. Mojtahedi D. Burr V. Gall V. Gibbs G. R.. (2021). Well-being programmes in prisons in England and Wales: a mixed-methods study. Int. J. Prison. Health 18, 259–274. doi: 10.1108/IJPH-03-2021-0021 34382757

[ref89] Veerman J. L. Dowrick C. Ayuso-Mateos J. L. Dunn G. Barendregt J. J. (2009). Population prevalence of depression and mean Beck depression inventory score. Br. J. Psychiatry 195, 516–519. doi: 10.1192/bjp.bp.109.066191, PMID: 19949201

[ref90] Violanti J. M. Charles L. E. McCanlies E. Hartley T. A. Baughman P. Andrew M. E. (2017). Police stressors and health: a state-of-the-art review. Policing 40, 642–656. doi: 10.1108/PIJPSM-06-2016-0097, PMID: 30846905 PMC6400077

[ref91] Violanti J. M. Slaven J. E. Charles L. E. Burchfiel C. M. Andrew M. E. Homish G. G. (2011). Police and alcohol use: a descriptive analysis and associations with stress outcomes. Am. J. Crim. Justice 36, 344–356. doi: 10.1007/s12103-011-9121-7

[ref92] Wainwright A. Mojtahedi D. (2020). An examination of stigmatising attributions about mental illness amongst police custody staff. Int. J. Law Psychiatry 68:101522. doi: 10.1016/j.ijlp.2019.101522, PMID: 32033693

[ref93] Wang H. Mo C. Fang F. (2020). Dissociated deficits in attentional networks in social anxiety and depression. Sci. China Life Sci. 63, 1071–1078. doi: 10.1007/s11427-019-1624-5, PMID: 32112270

[ref94] Wild J. Greenberg N. Moulds M. L.. (2020). Pre-incident training to build resilience in first responders: recommendations on what to and what not to do. Psychiatry 83, 128–142. doi: 10.1080/00332747.2020.1750215, PMID: 32338579

[ref96] Woodhams J. Duran F. (2024). A model for secondary traumatic stress following workplace exposure to traumatic material in analytical staff. Commun. Psychol. 2:13. doi: 10.1038/s44271-024-00060-1 39242898 PMC11332005

[ref97] World Health Organisation (2020) Burnout an ‘occupational phenomenon’: International Classification of Diseases. Available online at: https://www.who.int/news/item/28-05-2019-burn-out-anoccupational-phenomenon-international-classification-of-diseases (accessed 17 September 2024).

